# (*S*)-α-Benzyl­prolinium *cis*-[(*S*)-α-benzyl­prolinato]dichloridopalladium(II)

**DOI:** 10.1107/S1600536813009525

**Published:** 2013-04-13

**Authors:** David B. Hobart, Joseph S. Merola

**Affiliations:** a Department of Chemistry, Virginia Tech, Blacksburg, VA 24061, USA

## Abstract

The title complex salt, (C_12_H_16_NO_2_)[PdCl_2_(C_12_H_14_NO_2_)], is of inter­est with respect to organic and organometallic catalysis. The compound crystallizes as very small orange–red irregular prisms and the asymmetric unit contains three crystallographically distinct cation–anion pairs. The coordination geometry about the palladium atoms is square-planar with the chloride ligands *cis* to one another. The structure displays N—H⋯Cl and O—H⋯O hydrogen bonding such that the N—H⋯Cl hydrogen bonds align the cation–anion pairs in a linear fashion along [001], with the O—H⋯O hydrogen bonds connecting these linear strands along [100] and [010].

## Related literature

For the use of benzyl­proline in organocatalysis, see: Sutar & Joshi (2013 ▶); Cordova *et al.* (2004 ▶); Rispens *et al.* (1995 ▶). For other mono-amino acid halide complexes of palladium(II), see: Akat’eva *et al.* (2004 ▶); Asanin *et al.* (2004 ▶); Chernova *et al.* (1976 ▶, 1978 ▶); Djuran & Milinkovic (1999 ▶, 2000 ▶); Faraglia *et al.* (1997 ▶); Hao *et al.* (2007 ▶, 2009 ▶); Krylova *et al.* (1994 ▶); Spacu & Ungureanu-Vicol (1966 ▶); Vicol & Harabor (1974 ▶).
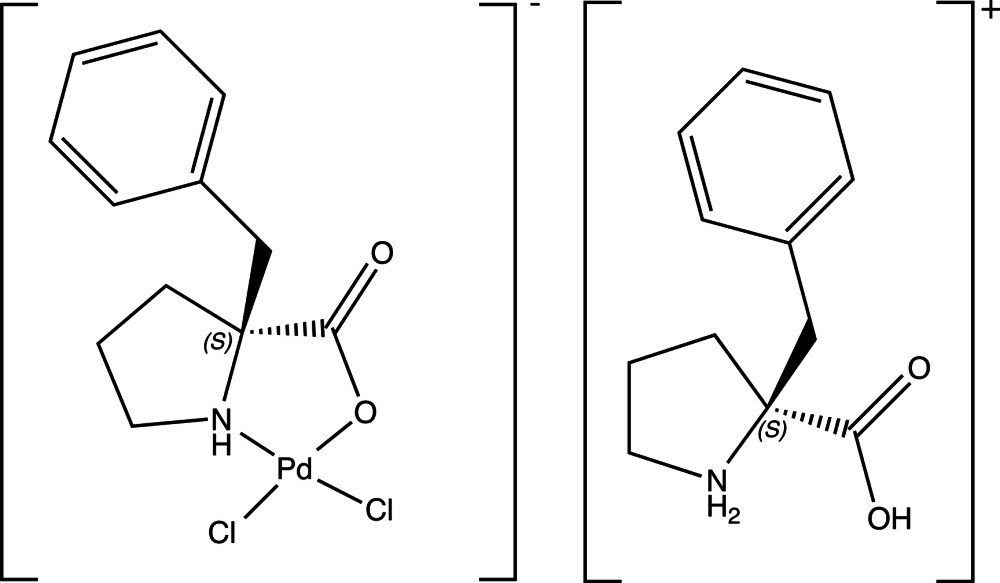


## Experimental

### 

#### Crystal data

(C_12_H_16_NO_2_)[PdCl_2_(C_12_H_14_NO_2_)]
*M* *_r_* = 587.80
Triclinic, 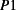

*a* = 10.6095 (5) Å
*b* = 12.3870 (4) Å
*c* = 14.6878 (4) Å
α = 101.376 (3)°
β = 93.421 (3)°
γ = 101.996 (3)°
*V* = 1840.80 (11) Å^3^
*Z* = 3
Mo *K*α radiation
μ = 1.01 mm^−1^
*T* = 100 K
0.19 × 0.12 × 0.11 mm

#### Data collection

Oxford Diffraction Gemini Ultra diffractometer
Absorption correction: gaussian (*CrysAlis PRO*; Agilent, 2013 ▶) *T*_min_ = 0.884, *T*_max_ = 0.929
40995 measured reflections
23898 independent reflections
21552 reflections with *I* > 2σ(*I*)
*R*_int_ = 0.033

#### Refinement

*R*[*F* ^2^ > 2σ(*F* ^2^)] = 0.039
*wR*(*F* ^2^) = 0.082
*S* = 1.03
23898 reflections
895 parameters
3 restraints
H-atom parameters constrained
Δρ_max_ = 1.99 e Å^−3^
Δρ_min_ = −0.77 e Å^−3^
Absolute structure: Flack (1983 ▶), 11561 Friedel pairs
Flack parameter: −0.039 (12)

### 

Data collection: *CrysAlis PRO* (Agilent, 2013 ▶); cell refinement: *CrysAlis PRO*; data reduction: *CrysAlis PRO*; program(s) used to solve structure: *SHELXS97* (Sheldrick, 2008 ▶); program(s) used to refine structure: *SHELXL97* (Sheldrick, 2008 ▶); molecular graphics: *OLEX2* (Dolomanov *et al.*, 2009 ▶); software used to prepare material for publication: *OLEX2*.

## Supplementary Material

Crystal structure: contains datablock(s) global, I. DOI: 10.1107/S1600536813009525/sj5316sup1.cif

Structure factors: contains datablock(s) I. DOI: 10.1107/S1600536813009525/sj5316Isup2.hkl

Additional supplementary materials:  crystallographic information; 3D view; checkCIF report

## Figures and Tables

**Table 1:** Hydrogen-bond geometry (Å, °)

*D*—H⋯*A*	*D*—H	H⋯*A*	*D*⋯*A*	*D*—H⋯*A*
O4—H4⋯O2^i^	0.84	1.69	2.521 (3)	171
O8—H8*A*⋯O6^ii^	0.84	1.71	2.547 (3)	171
O12—H12*A*⋯O10^iii^	0.84	1.73	2.565 (3)	177
N1—H1⋯Cl3	0.93	2.46	3.334 (3)	156
N2—H2*C*⋯Cl6^iv^	0.92	2.45	3.249 (3)	145
N3—H3⋯Cl2	0.93	2.48	3.354 (3)	156
N4—H4*A*⋯Cl3	0.92	2.35	3.216 (3)	156
N5—H5⋯Cl4	0.93	2.44	3.319 (3)	159
N6—H6*D*⋯Cl1^v^	0.92	2.37	3.235 (3)	156

Symmetry codes: (i) 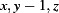; (ii) 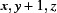; (iii) 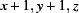; (iv) 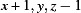; (v) 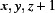.

**Fig. 1.**
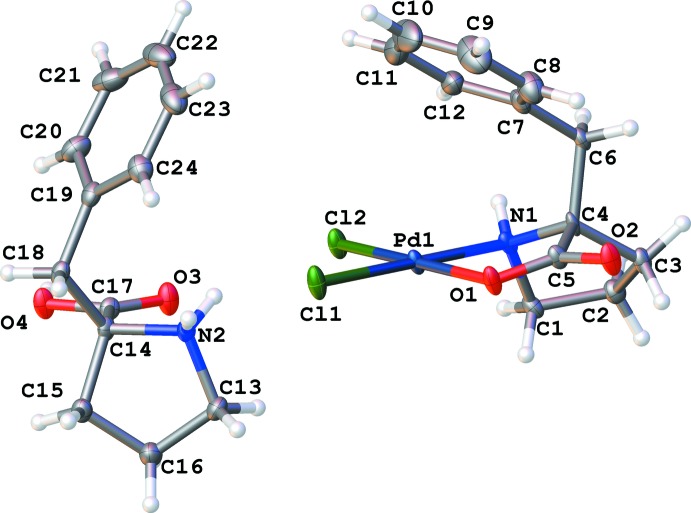
A view of a single cation/anion pair of the title compound showing atom labels. Displacement ellipsoids are drawn at the 50% probability level.

**Fig. 2.**
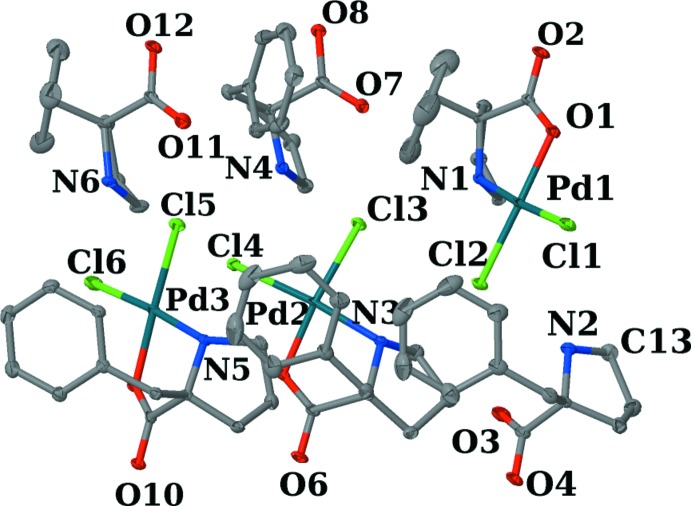
View of the unit cell contents of the title compound along [100] showing displacement ellipsoids at the 50% probability level.

**Fig. 3.**
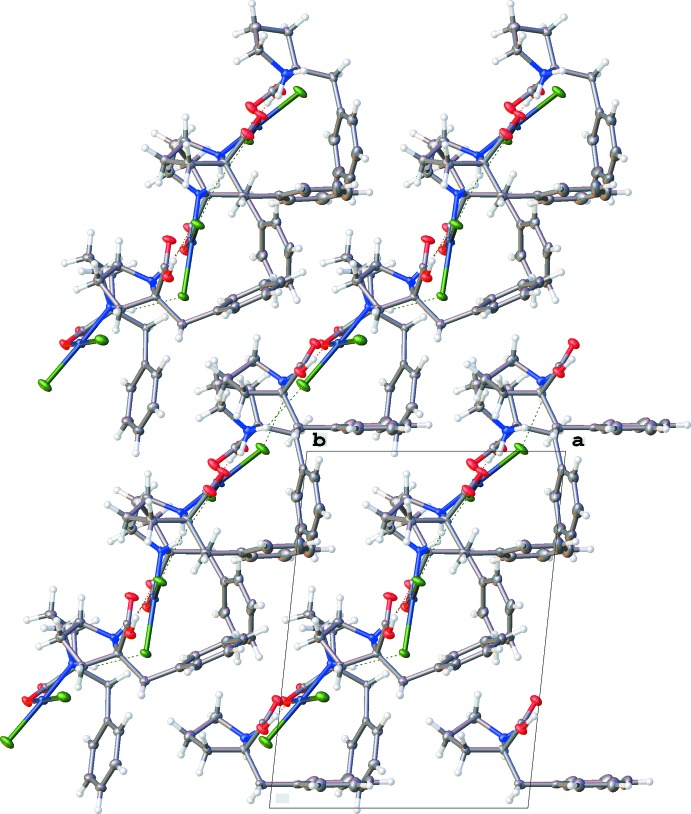
A view of a section of the crystal packing diagram of the title compound along [010] showing N1–H1···Cl3i, N3–H3···Cl2ii, N4–H4A···Cl3iii, and N5–H5···Cl4iv hydrogen bonds [Symmetry codes: (i) -1+x, -1+y, +z; (ii) -1+x, -1+y, +z; (iii) -1+x, -1+y, +z; (iv)-1+x, -1+y, +z].

## supplementary crystallographic information

### Comment

The title compound was isolated as a minor product from a reaction mixture of palladium(II) acetate and S-(α)-benzylproline hydrochloride. The expected product was the bis-amino acid palladium(II) chelate and was indeed formed in good yield. Attempts to crystallize the expected product were successful, however in every instance very small orange-red crystals of the title compound were observed to form along with the expected product.

### Experimental

The title compound was isolated as one of two products from a reaction mixture of 0.22 mmol palladium(II) acetate and 0.49 mmol *S*-(*a*)-benzylproline hydrochloride in 3 ml of 50/50 (*v*/*v*) acetone/water. Single crystals suitable for diffraction were grown *via* slow evaporation from a 50/50 (*v*/*v*) solution of acetone and water.

### Refinement

Refinement of F^2^ against ALL reflections. The weighted *R*-factor *wR* and goodness of fit S are based on F^2^, conventional *R*-factors *R* are based on F, with F set to zero for negative F^2^. The threshold expression of F^2^ > 2sigma(F^2^) is used only for calculating *R*-factors(gt) *etc*. and is not relevant to the choice of reflections for refinement. *R*-factors based on F^2^ are statistically about twice as large as those based on F, and R– factors based on ALL data will be even larger.

### Crystal data

**Table:**

(C_12_H_16_NO_2_)[PdCl_2_(C_12_H_14_NO_2_)]	*Z* = 3
*M**_r_* = 587.80	*F*(000) = 900
Triclinic, *P*1	*D*_x_ = 1.591 Mg m^−^^3^
*a* = 10.6095 (5) Å	Mo *K*α radiation, λ = 0.71073 Å
*b* = 12.3870 (4) Å	Cell parameters from 16328 reflections
*c* = 14.6878 (4) Å	θ = 3.4–32.6°
α = 101.376 (3)°	µ = 1.01 mm^−^^1^
β = 93.421 (3)°	*T* = 100 K
γ = 101.996 (3)°	Irregular, clear orange red
*V* = 1840.80 (11) Å^3^	0.19 × 0.12 × 0.11 mm

### Data collection

**Table:**

Oxford Diffraction Gemini Ultra diffractometer	23898 independent reflections
Radiation source: fine-focus sealed tube, fine-focus sealed tube	21552 reflections with *I* > 2σ(*I*)
Graphite monochromator	*R*_int_ = 0.033
Detector resolution: 16.0122 pixels mm^-1^	θ_max_ = 32.6°, θ_min_ = 3.4°
φ and ω scans	*h* = −15→15
Absorption correction: gaussian (*CrysAlis PRO*; Agilent, 2013)	*k* = −18→18
*T*_min_ = 0.884, *T*_max_ = 0.929	*l* = −22→21
40995 measured reflections	

### Refinement

**Table:**

Refinement on *F*^2^	Secondary atom site location: difference Fourier map
Least-squares matrix: full	Hydrogen site location: inferred from neighbouring sites
*R*[*F*^2^ > 2σ(*F*^2^)] = 0.039	H-atom parameters constrained
*wR*(*F*^2^) = 0.082	*w* = 1/[σ^2^(*F*_o_^2^) + (0.0338*P*)^2^] where *P* = (*F*_o_^2^ + 2*F*_c_^2^)/3
*S* = 1.03	(Δ/σ)_max_ = 0.001
23898 reflections	Δρ_max_ = 1.99 e Å^−^^3^
895 parameters	Δρ_min_ = −0.77 e Å^−^^3^
3 restraints	Absolute structure: Flack (1983), 11561 Friedel pairs
Primary atom site location: structure-invariant direct methods	Flack parameter: −0.039 (12)

### Special details

**Table:**

Geometry. All e.s.d.'s (except the e.s.d. in the dihedral angle between two l.s. planes) are estimated using the full covariance matrix. The cell e.s.d.'s are taken into account individually in the estimation of e.s.d.'s in distances, angles and torsion angles; correlations between e.s.d.'s in cell parameters are only used when they are defined by crystal symmetry. An approximate (isotropic) treatment of cell e.s.d.'s is used for estimating e.s.d.'s involving l.s. planes.
Refinement. Refinement of *F*^2^ against ALL reflections. The weighted *R*-factor *wR* and goodness of fit *S* are based on *F*^2^, conventional *R*-factors *R* are based on *F*, with *F* set to zero for negative *F*^2^. The threshold expression of *F*^2^ > σ(*F*^2^) is used only for calculating *R*-factors(gt) *etc*. and is not relevant to the choice of reflections for refinement. *R*-factors based on *F*^2^ are statistically about twice as large as those based on *F*, and *R*- factors based on ALL data will be even larger.

### Fractional atomic coordinates and isotropic or equivalent isotropic displacement parameters (Å^2^)

**Table:**

	*x*	*y*	*z*	*U*_iso_*/*U*_eq_	
Pd1	0.67261 (2)	0.638283 (17)	0.088258 (15)	0.01431 (5)	
Pd2	0.46952 (2)	0.423409 (17)	0.410899 (15)	0.01132 (5)	
Pd3	0.08081 (2)	0.400908 (17)	0.697611 (16)	0.01251 (5)	
Cl1	0.82733 (9)	0.59507 (7)	−0.00703 (6)	0.02285 (18)	
Cl2	0.64122 (9)	0.46476 (7)	0.12617 (6)	0.01968 (18)	
Cl3	0.48504 (9)	0.59104 (7)	0.36423 (6)	0.01971 (17)	
Cl4	0.45906 (8)	0.50373 (6)	0.56625 (5)	0.01566 (14)	
Cl5	0.16046 (10)	0.58439 (7)	0.68811 (6)	0.02175 (18)	
Cl6	−0.04104 (10)	0.45607 (7)	0.81541 (6)	0.02395 (19)	
O1	0.6849 (3)	0.7922 (2)	0.05762 (17)	0.0192 (5)	
O2	0.6409 (3)	0.9599 (2)	0.11048 (18)	0.0211 (5)	
O3	0.6453 (2)	0.1864 (2)	0.03745 (17)	0.0228 (5)	
O4	0.7477 (3)	0.0493 (2)	−0.01369 (17)	0.0200 (5)	
H4	0.7052	0.0164	0.0234	0.030*	
O5	0.4467 (2)	0.26793 (19)	0.43794 (16)	0.0160 (5)	
O6	0.4588 (3)	0.09164 (19)	0.37613 (17)	0.0221 (5)	
O7	0.3816 (3)	0.84199 (19)	0.40754 (16)	0.0208 (5)	
O8	0.3906 (3)	1.00261 (19)	0.51286 (16)	0.0180 (5)	
H8A	0.4052	1.0333	0.4673	0.027*	
O9	0.0186 (2)	0.23703 (18)	0.70130 (16)	0.0159 (5)	
O10	0.0735 (2)	0.07173 (18)	0.66413 (15)	0.0145 (4)	
O11	0.9879 (2)	0.81848 (19)	0.69815 (16)	0.0200 (5)	
O12	0.9414 (2)	0.97655 (19)	0.77902 (17)	0.0190 (5)	
H12A	0.9867	1.0080	0.7425	0.028*	
N1	0.5421 (3)	0.6887 (2)	0.17129 (18)	0.0144 (5)	
H1	0.5533	0.6658	0.2273	0.017*	
C13	0.6473 (3)	0.3179 (3)	−0.1260 (2)	0.0199 (6)	
H13A	0.6646	0.3764	−0.1637	0.024*	
H13B	0.5702	0.3263	−0.0928	0.024*	
N3	0.4768 (3)	0.3397 (2)	0.27965 (18)	0.0123 (5)	
H3	0.5412	0.3824	0.2529	0.015*	
N4	0.3312 (3)	0.7084 (2)	0.52338 (18)	0.0142 (5)	
H4A	0.3868	0.6974	0.4785	0.017*	
H4B	0.3464	0.6687	0.5681	0.017*	
N5	0.1816 (3)	0.3382 (2)	0.59457 (18)	0.0126 (5)	
H5	0.2646	0.3841	0.6035	0.015*	
N6	0.8830 (3)	0.6868 (2)	0.80445 (19)	0.0176 (6)	
H6C	0.9516	0.6791	0.7702	0.021*	
H6D	0.8904	0.6542	0.8550	0.021*	
C1	0.4041 (3)	0.6449 (3)	0.1320 (2)	0.0176 (6)	
H1A	0.3709	0.5679	0.1424	0.021*	
H1B	0.3940	0.6428	0.0642	0.021*	
C2	0.3338 (3)	0.7281 (3)	0.1850 (2)	0.0190 (6)	
H2A	0.3173	0.7123	0.2472	0.023*	
H2B	0.2504	0.7257	0.1498	0.023*	
C3	0.4282 (3)	0.8428 (3)	0.1939 (2)	0.0168 (6)	
H3A	0.4217	0.8949	0.2531	0.020*	
H3B	0.4092	0.8778	0.1411	0.020*	
C4	0.5650 (3)	0.8169 (3)	0.1925 (2)	0.0141 (6)	
C5	0.6351 (3)	0.8593 (3)	0.1147 (2)	0.0156 (6)	
C6	0.6505 (3)	0.8689 (3)	0.2858 (2)	0.0157 (6)	
H6A	0.6485	0.9498	0.3039	0.019*	
H6B	0.6126	0.8313	0.3346	0.019*	
C7	0.7903 (3)	0.8593 (3)	0.2838 (2)	0.0213 (7)	
C8	0.8837 (4)	0.9489 (3)	0.2675 (3)	0.0319 (9)	
H8	0.8596	1.0162	0.2589	0.038*	
C9	1.0122 (4)	0.9401 (4)	0.2639 (4)	0.0444 (12)	
H9	1.0756	1.0014	0.2530	0.053*	
C10	1.0474 (4)	0.8421 (5)	0.2761 (3)	0.0472 (13)	
H10	1.1352	0.8365	0.2744	0.057*	
C11	0.9558 (4)	0.7531 (4)	0.2908 (3)	0.0377 (10)	
H11	0.9801	0.6854	0.2981	0.045*	
C12	0.8278 (4)	0.7614 (3)	0.2949 (3)	0.0270 (8)	
H12	0.7652	0.6994	0.3055	0.032*	
N2	0.7643 (3)	0.3282 (2)	−0.05704 (19)	0.0152 (5)	
H2C	0.8321	0.3831	−0.0665	0.018*	
H2D	0.7441	0.3482	0.0031	0.018*	
C14	0.8023 (3)	0.2156 (3)	−0.0716 (2)	0.0143 (6)	
C15	0.7538 (3)	0.1640 (3)	−0.1752 (2)	0.0182 (6)	
H15A	0.8160	0.1944	−0.2165	0.022*	
H15B	0.7401	0.0806	−0.1885	0.022*	
C16	0.6262 (4)	0.1998 (3)	−0.1879 (2)	0.0229 (7)	
H16A	0.5548	0.1469	−0.1683	0.027*	
H16B	0.6048	0.2015	−0.2541	0.027*	
C17	0.7219 (3)	0.1483 (3)	−0.0092 (2)	0.0145 (6)	
C18	0.9478 (3)	0.2305 (3)	−0.0477 (2)	0.0165 (6)	
H18A	0.9692	0.1553	−0.0577	0.020*	
H18B	0.9950	0.2731	−0.0904	0.020*	
C19	0.9928 (3)	0.2926 (3)	0.0522 (2)	0.0190 (6)	
C20	0.9786 (4)	0.2360 (3)	0.1259 (3)	0.0267 (8)	
H20	0.9481	0.1562	0.1128	0.032*	
C21	1.0084 (4)	0.2950 (4)	0.2175 (3)	0.0353 (10)	
H21	0.9974	0.2558	0.2669	0.042*	
C22	1.0544 (4)	0.4109 (4)	0.2374 (3)	0.0378 (10)	
H22	1.0738	0.4513	0.3005	0.045*	
C23	1.0720 (4)	0.4681 (3)	0.1661 (3)	0.0334 (9)	
H23	1.1058	0.5475	0.1800	0.040*	
C24	1.0403 (4)	0.4094 (3)	0.0732 (3)	0.0244 (7)	
H24	1.0511	0.4493	0.0241	0.029*	
C25	0.3532 (3)	0.3140 (3)	0.2181 (2)	0.0194 (7)	
H25A	0.3428	0.3799	0.1921	0.023*	
H25B	0.2779	0.2914	0.2524	0.023*	
C26	0.3674 (4)	0.2162 (3)	0.1413 (2)	0.0256 (7)	
H26A	0.2818	0.1733	0.1090	0.031*	
H26B	0.4225	0.2437	0.0948	0.031*	
C27	0.4316 (3)	0.1438 (3)	0.1933 (2)	0.0165 (6)	
H27A	0.4896	0.1067	0.1539	0.020*	
H27B	0.3655	0.0848	0.2113	0.020*	
C28	0.5096 (3)	0.2261 (2)	0.2806 (2)	0.0133 (6)	
C29	0.4682 (3)	0.1925 (3)	0.3713 (2)	0.0146 (6)	
C30	0.6562 (3)	0.2364 (3)	0.2815 (2)	0.0159 (6)	
H30A	0.6750	0.1612	0.2806	0.019*	
H30B	0.6845	0.2599	0.2240	0.019*	
C31	0.7341 (3)	0.3200 (3)	0.3654 (2)	0.0170 (6)	
C32	0.7869 (3)	0.4312 (3)	0.3596 (3)	0.0218 (7)	
H32	0.7800	0.4526	0.3012	0.026*	
C33	0.8489 (3)	0.5100 (3)	0.4371 (3)	0.0303 (9)	
H33	0.8853	0.5850	0.4316	0.036*	
C34	0.8586 (4)	0.4813 (4)	0.5227 (3)	0.0336 (9)	
H34	0.8989	0.5368	0.5764	0.040*	
C35	0.8090 (4)	0.3709 (4)	0.5301 (3)	0.0390 (11)	
H35	0.8172	0.3503	0.5888	0.047*	
C36	0.7469 (3)	0.2896 (3)	0.4511 (3)	0.0263 (8)	
H36	0.7137	0.2139	0.4561	0.032*	
C37	0.1922 (3)	0.6682 (3)	0.4799 (2)	0.0183 (6)	
H37A	0.1520	0.5951	0.4950	0.022*	
H37B	0.1876	0.6587	0.4112	0.022*	
C38	0.1243 (3)	0.7601 (3)	0.5223 (2)	0.0208 (7)	
H38A	0.0439	0.7270	0.5471	0.025*	
H38B	0.1017	0.8016	0.4748	0.025*	
C39	0.2203 (3)	0.8389 (3)	0.6007 (2)	0.0148 (6)	
H39A	0.2114	0.8129	0.6601	0.018*	
H39B	0.2072	0.9170	0.6101	0.018*	
C40	0.3523 (3)	0.8328 (3)	0.5672 (2)	0.0120 (6)	
C41	0.3775 (3)	0.8929 (3)	0.4858 (2)	0.0140 (6)	
C42	0.4657 (3)	0.8723 (3)	0.6432 (2)	0.0169 (6)	
H42A	0.4589	0.9454	0.6822	0.020*	
H42B	0.4596	0.8168	0.6838	0.020*	
C43	0.5970 (3)	0.8866 (3)	0.6057 (2)	0.0165 (6)	
C44	0.6633 (3)	0.9930 (3)	0.5974 (2)	0.0208 (7)	
H44	0.6286	1.0572	0.6185	0.025*	
C45	0.7794 (4)	1.0057 (3)	0.5586 (3)	0.0258 (8)	
H45	0.8247	1.0789	0.5541	0.031*	
C46	0.8306 (3)	0.9130 (3)	0.5260 (2)	0.0249 (7)	
H46	0.9098	0.9219	0.4983	0.030*	
C47	0.7649 (4)	0.8073 (3)	0.5345 (3)	0.0232 (7)	
H47	0.7996	0.7433	0.5128	0.028*	
C48	0.6497 (3)	0.7938 (3)	0.5740 (2)	0.0191 (6)	
H48	0.6059	0.7207	0.5796	0.023*	
C49	0.1263 (3)	0.3306 (3)	0.4962 (2)	0.0160 (6)	
H49A	0.1940	0.3630	0.4595	0.019*	
H49B	0.0554	0.3714	0.4957	0.019*	
C50	0.0754 (3)	0.2050 (3)	0.4566 (2)	0.0188 (6)	
H50A	−0.0135	0.1790	0.4728	0.023*	
H50B	0.0751	0.1869	0.3878	0.023*	
C51	0.1708 (3)	0.1518 (2)	0.5033 (2)	0.0142 (6)	
H51A	0.2525	0.1587	0.4735	0.017*	
H51B	0.1336	0.0710	0.5011	0.017*	
C52	0.1932 (3)	0.2208 (2)	0.6037 (2)	0.0117 (6)	
C53	0.0868 (3)	0.1721 (3)	0.6605 (2)	0.0114 (5)	
C54	0.3267 (3)	0.2258 (2)	0.6549 (2)	0.0125 (5)	
H54A	0.3389	0.1480	0.6508	0.015*	
H54B	0.3958	0.2662	0.6235	0.015*	
C55	0.3391 (3)	0.2852 (3)	0.7567 (2)	0.0133 (5)	
C56	0.3866 (3)	0.4017 (3)	0.7852 (2)	0.0160 (6)	
H56	0.4186	0.4444	0.7411	0.019*	
C57	0.3874 (3)	0.4556 (3)	0.8775 (2)	0.0199 (7)	
H57	0.4207	0.5348	0.8963	0.024*	
C58	0.3399 (3)	0.3946 (3)	0.9425 (2)	0.0214 (7)	
H58	0.3390	0.4320	1.0054	0.026*	
C59	0.2937 (4)	0.2784 (3)	0.9148 (2)	0.0219 (7)	
H59	0.2612	0.2361	0.9591	0.026*	
C60	0.2946 (3)	0.2240 (3)	0.8233 (2)	0.0173 (6)	
H60	0.2648	0.1443	0.8055	0.021*	
C61	0.7578 (4)	0.6322 (3)	0.7458 (3)	0.0221 (7)	
H61A	0.7371	0.5494	0.7408	0.027*	
H61B	0.7599	0.6480	0.6824	0.027*	
C62	0.6624 (3)	0.6863 (3)	0.7985 (2)	0.0225 (7)	
H62A	0.6363	0.6490	0.8503	0.027*	
H62B	0.5840	0.6823	0.7567	0.027*	
C63	0.7358 (3)	0.8099 (3)	0.8362 (2)	0.0188 (6)	
H63A	0.7149	0.8377	0.9002	0.023*	
H63B	0.7114	0.8589	0.7956	0.023*	
C64	0.8815 (3)	0.8110 (2)	0.8366 (2)	0.0144 (6)	
C65	0.9438 (3)	0.8686 (3)	0.7632 (2)	0.0142 (6)	
C66	0.9599 (3)	0.8611 (3)	0.9333 (2)	0.0178 (6)	
H66A	0.9471	0.9385	0.9559	0.021*	
H66B	0.9250	0.8149	0.9778	0.021*	
C67	1.1026 (3)	0.8664 (3)	0.9335 (2)	0.0188 (6)	
C68	1.1891 (4)	0.9682 (3)	0.9352 (3)	0.0260 (8)	
H68	1.1568	1.0346	0.9372	0.031*	
C69	1.3211 (4)	0.9754 (4)	0.9340 (3)	0.0337 (9)	
H69	1.3784	1.0463	0.9370	0.040*	
C70	1.3692 (4)	0.8781 (4)	0.9283 (3)	0.0366 (10)	
H70	1.4593	0.8818	0.9259	0.044*	
C71	1.2853 (4)	0.7771 (4)	0.9264 (3)	0.0388 (10)	
H71	1.3176	0.7105	0.9223	0.047*	
C72	1.1531 (4)	0.7712 (3)	0.9302 (3)	0.0313 (9)	
H72	1.0967	0.7008	0.9307	0.038*	

### Atomic displacement parameters (Å^2^)

**Table:**

	*U*^11^	*U*^22^	*U*^33^	*U*^12^	*U*^13^	*U*^23^
Pd1	0.02410 (13)	0.00915 (10)	0.01240 (10)	0.00584 (9)	0.00917 (9)	0.00436 (8)
Pd2	0.01530 (11)	0.00823 (10)	0.01248 (10)	0.00385 (8)	0.00656 (8)	0.00431 (8)
Pd3	0.01676 (11)	0.00978 (10)	0.01312 (10)	0.00479 (9)	0.00536 (9)	0.00444 (8)
Cl1	0.0365 (5)	0.0141 (3)	0.0248 (4)	0.0114 (3)	0.0196 (4)	0.0096 (3)
Cl2	0.0323 (5)	0.0113 (3)	0.0202 (4)	0.0082 (3)	0.0137 (3)	0.0080 (3)
Cl3	0.0349 (5)	0.0120 (3)	0.0172 (4)	0.0100 (3)	0.0126 (3)	0.0069 (3)
Cl4	0.0228 (4)	0.0113 (3)	0.0139 (3)	0.0033 (3)	0.0073 (3)	0.0040 (3)
Cl5	0.0346 (5)	0.0123 (4)	0.0204 (4)	0.0062 (3)	0.0077 (4)	0.0061 (3)
Cl6	0.0367 (5)	0.0166 (4)	0.0263 (4)	0.0144 (4)	0.0198 (4)	0.0091 (3)
O1	0.0339 (15)	0.0129 (11)	0.0158 (12)	0.0102 (11)	0.0138 (11)	0.0067 (9)
O2	0.0343 (15)	0.0108 (11)	0.0236 (12)	0.0072 (10)	0.0168 (11)	0.0097 (9)
O3	0.0295 (14)	0.0172 (11)	0.0276 (13)	0.0092 (10)	0.0159 (11)	0.0106 (10)
O4	0.0299 (14)	0.0134 (11)	0.0224 (13)	0.0074 (10)	0.0151 (11)	0.0109 (10)
O5	0.0235 (13)	0.0108 (11)	0.0161 (11)	0.0042 (9)	0.0080 (10)	0.0067 (9)
O6	0.0391 (15)	0.0099 (10)	0.0225 (12)	0.0090 (10)	0.0149 (11)	0.0084 (9)
O7	0.0358 (14)	0.0141 (11)	0.0138 (10)	0.0052 (10)	0.0088 (10)	0.0045 (9)
O8	0.0266 (13)	0.0126 (11)	0.0162 (11)	0.0055 (10)	0.0063 (10)	0.0040 (9)
O9	0.0199 (12)	0.0109 (10)	0.0194 (11)	0.0048 (9)	0.0107 (9)	0.0052 (9)
O10	0.0179 (11)	0.0102 (10)	0.0172 (11)	0.0035 (8)	0.0060 (9)	0.0057 (8)
O11	0.0254 (12)	0.0184 (11)	0.0201 (11)	0.0089 (10)	0.0099 (10)	0.0066 (9)
O12	0.0274 (13)	0.0122 (10)	0.0211 (12)	0.0061 (9)	0.0122 (10)	0.0082 (9)
N1	0.0242 (14)	0.0088 (11)	0.0116 (12)	0.0039 (10)	0.0064 (10)	0.0041 (9)
C13	0.0188 (16)	0.0210 (16)	0.0224 (16)	0.0092 (13)	0.0007 (13)	0.0056 (13)
N3	0.0141 (12)	0.0083 (11)	0.0157 (12)	0.0030 (9)	0.0060 (10)	0.0039 (9)
N4	0.0186 (13)	0.0115 (12)	0.0137 (12)	0.0042 (10)	0.0055 (10)	0.0035 (10)
N5	0.0157 (13)	0.0086 (11)	0.0135 (12)	0.0011 (9)	0.0022 (10)	0.0040 (9)
N6	0.0271 (15)	0.0085 (12)	0.0177 (13)	0.0031 (11)	0.0027 (11)	0.0048 (10)
C1	0.0216 (16)	0.0145 (14)	0.0148 (14)	0.0023 (12)	0.0043 (12)	−0.0004 (11)
C2	0.0225 (17)	0.0152 (14)	0.0190 (15)	0.0036 (13)	0.0043 (13)	0.0033 (12)
C3	0.0242 (17)	0.0107 (13)	0.0178 (15)	0.0056 (12)	0.0085 (13)	0.0047 (11)
C4	0.0245 (17)	0.0083 (13)	0.0118 (14)	0.0071 (12)	0.0084 (12)	0.0018 (11)
C5	0.0236 (17)	0.0104 (13)	0.0142 (14)	0.0040 (12)	0.0063 (12)	0.0047 (11)
C6	0.0230 (16)	0.0104 (13)	0.0134 (14)	0.0047 (12)	0.0052 (12)	−0.0001 (11)
C7	0.0242 (17)	0.0207 (16)	0.0172 (15)	0.0074 (14)	0.0047 (13)	−0.0032 (13)
C8	0.028 (2)	0.0283 (19)	0.036 (2)	0.0050 (16)	0.0089 (17)	0.0009 (17)
C9	0.026 (2)	0.045 (3)	0.053 (3)	−0.0011 (19)	0.011 (2)	−0.004 (2)
C10	0.027 (2)	0.069 (3)	0.042 (3)	0.023 (2)	0.0043 (19)	−0.010 (2)
C11	0.040 (2)	0.046 (3)	0.030 (2)	0.026 (2)	0.0016 (18)	0.0003 (19)
C12	0.030 (2)	0.031 (2)	0.0209 (17)	0.0148 (17)	−0.0001 (15)	−0.0001 (15)
N2	0.0172 (13)	0.0109 (12)	0.0183 (13)	0.0040 (10)	0.0028 (10)	0.0036 (10)
C14	0.0170 (15)	0.0097 (13)	0.0186 (15)	0.0047 (12)	0.0052 (12)	0.0060 (12)
C15	0.0215 (16)	0.0188 (15)	0.0168 (15)	0.0076 (13)	0.0051 (12)	0.0056 (12)
C16	0.0272 (18)	0.0222 (17)	0.0202 (16)	0.0100 (14)	0.0006 (14)	0.0027 (13)
C17	0.0153 (15)	0.0113 (13)	0.0175 (15)	0.0019 (11)	0.0029 (12)	0.0052 (11)
C18	0.0149 (15)	0.0183 (15)	0.0200 (15)	0.0071 (12)	0.0063 (12)	0.0080 (12)
C19	0.0135 (14)	0.0192 (15)	0.0261 (17)	0.0062 (12)	0.0034 (12)	0.0064 (13)
C20	0.0235 (18)	0.0311 (19)	0.0286 (19)	0.0063 (15)	0.0007 (14)	0.0141 (16)
C21	0.025 (2)	0.059 (3)	0.0248 (19)	0.0110 (19)	−0.0013 (15)	0.0135 (19)
C22	0.0225 (19)	0.056 (3)	0.027 (2)	0.0095 (19)	−0.0019 (16)	−0.0070 (19)
C23	0.0233 (19)	0.029 (2)	0.041 (2)	0.0032 (16)	0.0020 (17)	−0.0073 (17)
C24	0.0211 (17)	0.0225 (17)	0.0286 (18)	0.0035 (14)	0.0057 (14)	0.0036 (14)
C25	0.0194 (16)	0.0199 (16)	0.0201 (16)	0.0083 (13)	0.0003 (13)	0.0034 (13)
C26	0.032 (2)	0.0216 (17)	0.0214 (17)	0.0046 (15)	0.0006 (15)	0.0041 (14)
C27	0.0216 (16)	0.0116 (13)	0.0155 (14)	0.0024 (12)	0.0052 (12)	0.0015 (11)
C28	0.0188 (15)	0.0105 (13)	0.0138 (14)	0.0057 (11)	0.0061 (11)	0.0061 (11)
C29	0.0179 (15)	0.0111 (13)	0.0164 (14)	0.0029 (11)	0.0070 (12)	0.0057 (11)
C30	0.0173 (15)	0.0159 (14)	0.0161 (14)	0.0057 (12)	0.0061 (12)	0.0042 (12)
C31	0.0140 (14)	0.0194 (15)	0.0199 (15)	0.0068 (12)	0.0023 (12)	0.0062 (12)
C32	0.0182 (16)	0.0209 (16)	0.0276 (18)	0.0080 (13)	0.0014 (14)	0.0047 (14)
C33	0.0167 (17)	0.0242 (18)	0.046 (2)	0.0075 (14)	−0.0025 (16)	−0.0038 (17)
C34	0.0182 (18)	0.046 (2)	0.029 (2)	0.0097 (17)	−0.0021 (15)	−0.0099 (18)
C35	0.024 (2)	0.073 (3)	0.0230 (19)	0.015 (2)	0.0010 (16)	0.013 (2)
C36	0.0198 (17)	0.036 (2)	0.0260 (18)	0.0099 (15)	0.0007 (14)	0.0106 (16)
C37	0.0206 (16)	0.0152 (15)	0.0175 (15)	0.0008 (12)	0.0028 (13)	0.0025 (12)
C38	0.0212 (16)	0.0212 (16)	0.0200 (15)	0.0061 (13)	0.0041 (13)	0.0022 (13)
C39	0.0192 (15)	0.0134 (14)	0.0133 (14)	0.0039 (12)	0.0068 (12)	0.0042 (11)
C40	0.0164 (14)	0.0097 (13)	0.0107 (13)	0.0029 (11)	0.0042 (11)	0.0035 (11)
C41	0.0165 (15)	0.0122 (14)	0.0153 (14)	0.0045 (11)	0.0058 (12)	0.0050 (11)
C42	0.0185 (15)	0.0175 (15)	0.0161 (14)	0.0037 (12)	0.0027 (12)	0.0072 (12)
C43	0.0163 (15)	0.0190 (15)	0.0141 (14)	0.0026 (12)	0.0010 (11)	0.0051 (12)
C44	0.0193 (16)	0.0160 (15)	0.0233 (16)	0.0011 (12)	−0.0021 (13)	−0.0004 (13)
C45	0.0197 (18)	0.0252 (18)	0.0307 (19)	−0.0026 (15)	0.0010 (15)	0.0108 (15)
C46	0.0160 (16)	0.0330 (19)	0.0230 (17)	0.0012 (14)	0.0018 (13)	0.0045 (15)
C47	0.0194 (17)	0.0223 (17)	0.0255 (17)	0.0072 (14)	−0.0012 (14)	−0.0023 (14)
C48	0.0206 (16)	0.0174 (15)	0.0190 (15)	0.0052 (13)	−0.0016 (12)	0.0036 (12)
C49	0.0192 (15)	0.0170 (14)	0.0147 (14)	0.0043 (12)	0.0046 (12)	0.0096 (12)
C50	0.0250 (17)	0.0184 (15)	0.0140 (14)	0.0053 (13)	0.0019 (12)	0.0053 (12)
C51	0.0181 (15)	0.0094 (13)	0.0156 (14)	0.0027 (11)	0.0051 (11)	0.0034 (11)
C52	0.0130 (14)	0.0084 (13)	0.0145 (14)	0.0014 (11)	0.0041 (11)	0.0049 (11)
C53	0.0119 (13)	0.0120 (13)	0.0099 (12)	0.0002 (10)	0.0029 (10)	0.0034 (10)
C54	0.0138 (14)	0.0112 (13)	0.0137 (13)	0.0041 (11)	0.0037 (11)	0.0037 (11)
C55	0.0118 (13)	0.0146 (14)	0.0148 (13)	0.0038 (11)	0.0031 (11)	0.0047 (11)
C56	0.0207 (16)	0.0112 (13)	0.0163 (14)	0.0032 (12)	0.0002 (12)	0.0043 (11)
C57	0.0247 (17)	0.0134 (14)	0.0202 (16)	0.0042 (13)	0.0003 (13)	0.0007 (12)
C58	0.0230 (17)	0.0252 (17)	0.0150 (15)	0.0056 (14)	0.0041 (13)	0.0012 (13)
C59	0.0252 (18)	0.0240 (17)	0.0153 (15)	0.0008 (14)	0.0022 (13)	0.0063 (13)
C60	0.0182 (15)	0.0157 (14)	0.0169 (14)	0.0005 (12)	−0.0001 (12)	0.0051 (12)
C61	0.0255 (18)	0.0116 (14)	0.0252 (17)	0.0000 (13)	−0.0023 (14)	0.0002 (13)
C62	0.0219 (17)	0.0197 (16)	0.0235 (17)	−0.0014 (13)	−0.0010 (13)	0.0066 (13)
C63	0.0182 (16)	0.0188 (15)	0.0197 (15)	0.0021 (12)	0.0078 (13)	0.0051 (12)
C64	0.0191 (15)	0.0070 (12)	0.0182 (14)	0.0019 (11)	0.0063 (12)	0.0049 (11)
C65	0.0164 (14)	0.0123 (13)	0.0159 (14)	0.0036 (11)	0.0053 (11)	0.0061 (11)
C66	0.0250 (17)	0.0116 (14)	0.0160 (15)	0.0005 (12)	0.0059 (13)	0.0035 (12)
C67	0.0270 (17)	0.0171 (15)	0.0105 (13)	0.0034 (13)	−0.0003 (12)	0.0009 (12)
C68	0.0248 (18)	0.0210 (17)	0.0323 (19)	0.0018 (14)	0.0029 (15)	0.0092 (15)
C69	0.027 (2)	0.034 (2)	0.039 (2)	0.0004 (17)	0.0017 (17)	0.0132 (18)
C70	0.025 (2)	0.051 (3)	0.034 (2)	0.0130 (19)	−0.0058 (17)	0.007 (2)
C71	0.041 (2)	0.034 (2)	0.041 (2)	0.0191 (19)	−0.0160 (19)	0.0010 (19)
C72	0.036 (2)	0.0191 (17)	0.036 (2)	0.0041 (16)	−0.0114 (17)	0.0058 (16)

### Geometric parameters (Å, º)

**Table:**

Pd1—Cl1	2.2986 (8)	C25—H25B	0.9900
Pd1—Cl2	2.2886 (8)	C25—C26	1.523 (5)
Pd1—O1	2.024 (2)	C26—H26A	0.9900
Pd1—N1	2.014 (3)	C26—H26B	0.9900
Pd2—Cl3	2.2894 (8)	C26—C27	1.521 (5)
Pd2—Cl4	2.3215 (7)	C27—H27A	0.9900
Pd2—O5	2.011 (2)	C27—H27B	0.9900
Pd2—N3	2.015 (3)	C27—C28	1.542 (4)
Pd3—Cl5	2.2891 (9)	C28—C29	1.534 (4)
Pd3—Cl6	2.3021 (9)	C28—C30	1.533 (4)
Pd3—O9	2.012 (2)	C30—H30A	0.9900
Pd3—N5	2.028 (3)	C30—H30B	0.9900
O1—C5	1.278 (4)	C30—C31	1.514 (4)
O2—C5	1.249 (4)	C31—C32	1.396 (5)
O3—C17	1.204 (4)	C31—C36	1.391 (5)
O4—H4	0.8400	C32—H32	0.9500
O4—C17	1.301 (4)	C32—C33	1.374 (5)
O5—C29	1.279 (4)	C33—H33	0.9500
O6—C29	1.249 (4)	C33—C34	1.376 (6)
O7—C41	1.205 (4)	C34—H34	0.9500
O8—H8A	0.8400	C34—C35	1.388 (7)
O8—C41	1.313 (4)	C35—H35	0.9500
O9—C53	1.278 (4)	C35—C36	1.402 (6)
O10—C53	1.234 (4)	C36—H36	0.9500
O11—C65	1.207 (4)	C37—H37A	0.9900
O12—H12A	0.8400	C37—H37B	0.9900
O12—C65	1.317 (4)	C37—C38	1.523 (5)
N1—H1	0.9300	C38—H38A	0.9900
N1—C1	1.488 (4)	C38—H38B	0.9900
N1—C4	1.521 (4)	C38—C39	1.524 (5)
C13—H13A	0.9900	C39—H39A	0.9900
C13—H13B	0.9900	C39—H39B	0.9900
C13—N2	1.522 (4)	C39—C40	1.524 (4)
C13—C16	1.527 (5)	C40—C41	1.537 (4)
N3—H3	0.9300	C40—C42	1.521 (4)
N3—C25	1.487 (4)	C42—H42A	0.9900
N3—C28	1.521 (4)	C42—H42B	0.9900
N4—H4A	0.9200	C42—C43	1.517 (4)
N4—H4B	0.9200	C43—C44	1.391 (5)
N4—C37	1.512 (4)	C43—C48	1.393 (5)
N4—C40	1.512 (4)	C44—H44	0.9500
N5—H5	0.9300	C44—C45	1.380 (5)
N5—C49	1.505 (4)	C45—H45	0.9500
N5—C52	1.516 (4)	C45—C46	1.384 (5)
N6—H6C	0.9200	C46—H46	0.9500
N6—H6D	0.9200	C46—C47	1.382 (5)
N6—C61	1.491 (5)	C47—H47	0.9500
N6—C64	1.521 (4)	C47—C48	1.377 (5)
C1—H1A	0.9900	C48—H48	0.9500
C1—H1B	0.9900	C49—H49A	0.9900
C1—C2	1.515 (4)	C49—H49B	0.9900
C2—H2A	0.9900	C49—C50	1.519 (4)
C2—H2B	0.9900	C50—H50A	0.9900
C2—C3	1.535 (4)	C50—H50B	0.9900
C3—H3A	0.9900	C50—C51	1.518 (4)
C3—H3B	0.9900	C51—H51A	0.9900
C3—C4	1.551 (5)	C51—H51B	0.9900
C4—C5	1.520 (4)	C51—C52	1.528 (4)
C4—C6	1.541 (5)	C52—C53	1.537 (4)
C6—H6A	0.9900	C52—C54	1.547 (4)
C6—H6B	0.9900	C54—H54A	0.9900
C6—C7	1.514 (5)	C54—H54B	0.9900
C7—C8	1.395 (5)	C54—C55	1.515 (4)
C7—C12	1.389 (5)	C55—C56	1.395 (4)
C8—H8	0.9500	C55—C60	1.397 (4)
C8—C9	1.393 (6)	C56—H56	0.9500
C9—H9	0.9500	C56—C57	1.386 (4)
C9—C10	1.382 (7)	C57—H57	0.9500
C10—H10	0.9500	C57—C58	1.385 (5)
C10—C11	1.371 (7)	C58—H58	0.9500
C11—H11	0.9500	C58—C59	1.389 (5)
C11—C12	1.387 (6)	C59—H59	0.9500
C12—H12	0.9500	C59—C60	1.381 (4)
N2—H2C	0.9200	C60—H60	0.9500
N2—H2D	0.9200	C61—H61A	0.9900
N2—C14	1.510 (4)	C61—H61B	0.9900
C14—C15	1.541 (5)	C61—C62	1.498 (5)
C14—C17	1.537 (4)	C62—H62A	0.9900
C14—C18	1.527 (5)	C62—H62B	0.9900
C15—H15A	0.9900	C62—C63	1.541 (5)
C15—H15B	0.9900	C63—H63A	0.9900
C15—C16	1.522 (5)	C63—H63B	0.9900
C16—H16A	0.9900	C63—C64	1.543 (5)
C16—H16B	0.9900	C64—C65	1.516 (4)
C18—H18A	0.9900	C64—C66	1.546 (5)
C18—H18B	0.9900	C66—H66A	0.9900
C18—C19	1.513 (5)	C66—H66B	0.9900
C19—C20	1.401 (5)	C66—C67	1.502 (5)
C19—C24	1.394 (5)	C67—C68	1.392 (5)
C20—H20	0.9500	C67—C72	1.387 (5)
C20—C21	1.382 (6)	C68—H68	0.9500
C21—H21	0.9500	C68—C69	1.386 (6)
C21—C22	1.383 (7)	C69—H69	0.9500
C22—H22	0.9500	C69—C70	1.393 (6)
C22—C23	1.377 (6)	C70—H70	0.9500
C23—H23	0.9500	C70—C71	1.370 (6)
C23—C24	1.399 (5)	C71—H71	0.9500
C24—H24	0.9500	C71—C72	1.395 (6)
C25—H25A	0.9900	C72—H72	0.9500
			
Cl2—Pd1—Cl1	91.05 (3)	C29—C28—C27	112.1 (2)
O1—Pd1—Cl1	93.45 (7)	C30—C28—C27	113.1 (2)
O1—Pd1—Cl2	175.09 (8)	C30—C28—C29	108.1 (3)
N1—Pd1—Cl1	175.58 (8)	O5—C29—C28	119.3 (3)
N1—Pd1—Cl2	93.22 (8)	O6—C29—O5	123.4 (3)
N1—Pd1—O1	82.33 (10)	O6—C29—C28	117.3 (3)
Cl3—Pd2—Cl4	93.56 (3)	C28—C30—H30A	108.9
O5—Pd2—Cl3	173.81 (8)	C28—C30—H30B	108.9
O5—Pd2—Cl4	91.87 (7)	H30A—C30—H30B	107.7
O5—Pd2—N3	82.76 (10)	C31—C30—C28	113.6 (3)
N3—Pd2—Cl3	91.81 (7)	C31—C30—H30A	108.9
N3—Pd2—Cl4	174.63 (7)	C31—C30—H30B	108.9
Cl5—Pd3—Cl6	91.58 (3)	C32—C31—C30	120.5 (3)
O9—Pd3—Cl5	176.70 (7)	C36—C31—C30	120.6 (3)
O9—Pd3—Cl6	91.65 (7)	C36—C31—C32	118.8 (3)
O9—Pd3—N5	83.36 (9)	C31—C32—H32	119.5
N5—Pd3—Cl5	93.41 (7)	C33—C32—C31	121.0 (3)
N5—Pd3—Cl6	175.01 (8)	C33—C32—H32	119.5
C5—O1—Pd1	114.3 (2)	C32—C33—H33	119.7
C17—O4—H4	109.5	C32—C33—C34	120.5 (4)
C29—O5—Pd2	114.85 (19)	C34—C33—H33	119.7
C41—O8—H8A	109.5	C33—C34—H34	120.2
C53—O9—Pd3	114.33 (19)	C33—C34—C35	119.7 (4)
C65—O12—H12A	109.5	C35—C34—H34	120.2
Pd1—N1—H1	108.3	C34—C35—H35	119.9
C1—N1—Pd1	115.27 (19)	C34—C35—C36	120.1 (4)
C1—N1—H1	108.3	C36—C35—H35	119.9
C1—N1—C4	106.4 (3)	C31—C36—C35	119.9 (4)
C4—N1—Pd1	110.01 (19)	C31—C36—H36	120.1
C4—N1—H1	108.3	C35—C36—H36	120.1
H13A—C13—H13B	108.8	N4—C37—H37A	110.7
N2—C13—H13A	110.7	N4—C37—H37B	110.7
N2—C13—H13B	110.7	N4—C37—C38	105.2 (3)
N2—C13—C16	105.1 (3)	H37A—C37—H37B	108.8
C16—C13—H13A	110.7	C38—C37—H37A	110.7
C16—C13—H13B	110.7	C38—C37—H37B	110.7
Pd2—N3—H3	108.7	C37—C38—H38A	110.6
C25—N3—Pd2	114.5 (2)	C37—C38—H38B	110.6
C25—N3—H3	108.7	C37—C38—C39	105.8 (3)
C25—N3—C28	105.7 (2)	H38A—C38—H38B	108.7
C28—N3—Pd2	110.33 (18)	C39—C38—H38A	110.6
C28—N3—H3	108.7	C39—C38—H38B	110.6
H4A—N4—H4B	108.5	C38—C39—H39A	111.0
C37—N4—H4A	110.2	C38—C39—H39B	111.0
C37—N4—H4B	110.2	C38—C39—C40	104.0 (3)
C37—N4—C40	107.6 (2)	H39A—C39—H39B	109.0
C40—N4—H4A	110.2	C40—C39—H39A	111.0
C40—N4—H4B	110.2	C40—C39—H39B	111.0
Pd3—N5—H5	107.9	N4—C40—C39	101.3 (2)
C49—N5—Pd3	116.2 (2)	N4—C40—C41	104.8 (2)
C49—N5—H5	107.9	N4—C40—C42	112.3 (3)
C49—N5—C52	107.9 (2)	C39—C40—C41	111.5 (3)
C52—N5—Pd3	108.81 (18)	C42—C40—C39	115.0 (3)
C52—N5—H5	107.9	C42—C40—C41	111.1 (2)
H6C—N6—H6D	108.6	O7—C41—O8	126.3 (3)
C61—N6—H6C	110.4	O7—C41—C40	122.1 (3)
C61—N6—H6D	110.4	O8—C41—C40	111.6 (3)
C61—N6—C64	106.5 (3)	C40—C42—H42A	108.9
C64—N6—H6C	110.4	C40—C42—H42B	108.9
C64—N6—H6D	110.4	H42A—C42—H42B	107.7
N1—C1—H1A	110.9	C43—C42—C40	113.5 (3)
N1—C1—H1B	110.9	C43—C42—H42A	108.9
N1—C1—C2	104.3 (2)	C43—C42—H42B	108.9
H1A—C1—H1B	108.9	C44—C43—C42	120.0 (3)
C2—C1—H1A	110.9	C44—C43—C48	118.7 (3)
C2—C1—H1B	110.9	C48—C43—C42	121.1 (3)
C1—C2—H2A	111.1	C43—C44—H44	119.8
C1—C2—H2B	111.1	C45—C44—C43	120.3 (3)
C1—C2—C3	103.4 (3)	C45—C44—H44	119.8
H2A—C2—H2B	109.0	C44—C45—H45	119.6
C3—C2—H2A	111.1	C44—C45—C46	120.8 (3)
C3—C2—H2B	111.1	C46—C45—H45	119.6
C2—C3—H3A	110.6	C45—C46—H46	120.5
C2—C3—H3B	110.6	C47—C46—C45	119.0 (3)
C2—C3—C4	105.5 (3)	C47—C46—H46	120.5
H3A—C3—H3B	108.8	C46—C47—H47	119.6
C4—C3—H3A	110.6	C48—C47—C46	120.8 (3)
C4—C3—H3B	110.6	C48—C47—H47	119.6
N1—C4—C3	105.4 (3)	C43—C48—H48	119.8
N1—C4—C6	110.8 (3)	C47—C48—C43	120.5 (3)
C5—C4—N1	108.0 (2)	C47—C48—H48	119.8
C5—C4—C3	111.3 (3)	N5—C49—H49A	110.8
C5—C4—C6	108.6 (3)	N5—C49—H49B	110.8
C6—C4—C3	112.6 (3)	N5—C49—C50	104.7 (2)
O1—C5—C4	119.5 (3)	H49A—C49—H49B	108.9
O2—C5—O1	123.3 (3)	C50—C49—H49A	110.8
O2—C5—C4	117.2 (3)	C50—C49—H49B	110.8
C4—C6—H6A	108.6	C49—C50—H50A	111.1
C4—C6—H6B	108.6	C49—C50—H50B	111.1
H6A—C6—H6B	107.6	H50A—C50—H50B	109.1
C7—C6—C4	114.5 (3)	C51—C50—C49	103.2 (3)
C7—C6—H6A	108.6	C51—C50—H50A	111.1
C7—C6—H6B	108.6	C51—C50—H50B	111.1
C8—C7—C6	119.9 (3)	C50—C51—H51A	111.2
C12—C7—C6	121.4 (3)	C50—C51—H51B	111.2
C12—C7—C8	118.7 (4)	C50—C51—C52	102.9 (2)
C7—C8—H8	119.9	H51A—C51—H51B	109.1
C9—C8—C7	120.3 (4)	C52—C51—H51A	111.2
C9—C8—H8	119.9	C52—C51—H51B	111.2
C8—C9—H9	120.0	N5—C52—C51	104.5 (2)
C10—C9—C8	119.9 (4)	N5—C52—C53	109.6 (2)
C10—C9—H9	120.0	N5—C52—C54	110.2 (2)
C9—C10—H10	119.9	C51—C52—C53	109.9 (2)
C11—C10—C9	120.1 (4)	C51—C52—C54	114.1 (3)
C11—C10—H10	119.9	C53—C52—C54	108.5 (2)
C10—C11—H11	119.9	O9—C53—C52	118.8 (3)
C10—C11—C12	120.3 (4)	O10—C53—O9	124.0 (3)
C12—C11—H11	119.9	O10—C53—C52	117.2 (3)
C7—C12—H12	119.7	C52—C54—H54A	109.2
C11—C12—C7	120.7 (4)	C52—C54—H54B	109.2
C11—C12—H12	119.7	H54A—C54—H54B	107.9
C13—N2—H2C	110.0	C55—C54—C52	112.1 (2)
C13—N2—H2D	110.0	C55—C54—H54A	109.2
H2C—N2—H2D	108.4	C55—C54—H54B	109.2
C14—N2—C13	108.6 (2)	C56—C55—C54	121.4 (3)
C14—N2—H2C	110.0	C56—C55—C60	118.6 (3)
C14—N2—H2D	110.0	C60—C55—C54	119.8 (3)
N2—C14—C15	102.4 (2)	C55—C56—H56	119.8
N2—C14—C17	105.7 (3)	C57—C56—C55	120.4 (3)
N2—C14—C18	111.3 (3)	C57—C56—H56	119.8
C17—C14—C15	110.0 (3)	C56—C57—H57	119.8
C18—C14—C15	114.4 (3)	C58—C57—C56	120.5 (3)
C18—C14—C17	112.3 (3)	C58—C57—H57	119.8
C14—C15—H15A	111.1	C57—C58—H58	120.3
C14—C15—H15B	111.1	C57—C58—C59	119.4 (3)
H15A—C15—H15B	109.0	C59—C58—H58	120.3
C16—C15—C14	103.5 (3)	C58—C59—H59	119.8
C16—C15—H15A	111.1	C60—C59—C58	120.4 (3)
C16—C15—H15B	111.1	C60—C59—H59	119.8
C13—C16—H16A	110.7	C55—C60—H60	119.7
C13—C16—H16B	110.7	C59—C60—C55	120.6 (3)
C15—C16—C13	105.5 (3)	C59—C60—H60	119.7
C15—C16—H16A	110.7	N6—C61—H61A	111.2
C15—C16—H16B	110.7	N6—C61—H61B	111.2
H16A—C16—H16B	108.8	N6—C61—C62	102.8 (3)
O3—C17—O4	126.1 (3)	H61A—C61—H61B	109.1
O3—C17—C14	121.8 (3)	C62—C61—H61A	111.2
O4—C17—C14	112.1 (3)	C62—C61—H61B	111.2
C14—C18—H18A	109.1	C61—C62—H62A	110.9
C14—C18—H18B	109.1	C61—C62—H62B	110.9
H18A—C18—H18B	107.9	C61—C62—C63	104.3 (3)
C19—C18—C14	112.4 (3)	H62A—C62—H62B	108.9
C19—C18—H18A	109.1	C63—C62—H62A	110.9
C19—C18—H18B	109.1	C63—C62—H62B	110.9
C20—C19—C18	121.0 (3)	C62—C63—H63A	110.4
C24—C19—C18	120.2 (3)	C62—C63—H63B	110.4
C24—C19—C20	118.5 (3)	C62—C63—C64	106.6 (3)
C19—C20—H20	119.7	H63A—C63—H63B	108.6
C21—C20—C19	120.7 (4)	C64—C63—H63A	110.4
C21—C20—H20	119.7	C64—C63—H63B	110.4
C20—C21—H21	119.9	N6—C64—C63	103.3 (2)
C20—C21—C22	120.2 (4)	N6—C64—C66	110.9 (3)
C22—C21—H21	119.9	C63—C64—C66	113.8 (3)
C21—C22—H22	119.9	C65—C64—N6	105.4 (2)
C23—C22—C21	120.2 (4)	C65—C64—C63	112.5 (3)
C23—C22—H22	119.9	C65—C64—C66	110.4 (3)
C22—C23—H23	120.0	O11—C65—O12	125.6 (3)
C22—C23—C24	120.0 (4)	O11—C65—C64	122.4 (3)
C24—C23—H23	120.0	O12—C65—C64	112.0 (3)
C19—C24—C23	120.3 (4)	C64—C66—H66A	108.7
C19—C24—H24	119.8	C64—C66—H66B	108.7
C23—C24—H24	119.8	H66A—C66—H66B	107.6
N3—C25—H25A	111.1	C67—C66—C64	114.1 (3)
N3—C25—H25B	111.1	C67—C66—H66A	108.7
N3—C25—C26	103.3 (3)	C67—C66—H66B	108.7
H25A—C25—H25B	109.1	C68—C67—C66	120.4 (3)
C26—C25—H25A	111.1	C72—C67—C66	122.0 (3)
C26—C25—H25B	111.1	C72—C67—C68	117.6 (4)
C25—C26—H26A	111.0	C67—C68—H68	119.1
C25—C26—H26B	111.0	C69—C68—C67	121.8 (4)
H26A—C26—H26B	109.0	C69—C68—H68	119.1
C27—C26—C25	103.7 (3)	C68—C69—H69	120.2
C27—C26—H26A	111.0	C68—C69—C70	119.7 (4)
C27—C26—H26B	111.0	C70—C69—H69	120.2
C26—C27—H27A	110.7	C69—C70—H70	120.4
C26—C27—H27B	110.7	C71—C70—C69	119.3 (4)
C26—C27—C28	105.4 (3)	C71—C70—H70	120.4
H27A—C27—H27B	108.8	C70—C71—H71	119.7
C28—C27—H27A	110.7	C70—C71—C72	120.7 (4)
C28—C27—H27B	110.7	C72—C71—H71	119.7
N3—C28—C27	105.7 (2)	C67—C72—C71	121.0 (4)
N3—C28—C29	107.4 (2)	C67—C72—H72	119.5
N3—C28—C30	110.3 (2)	C71—C72—H72	119.5

### Hydrogen-bond geometry (Å, º)

**Table:**

*D*—H···*A*	*D*—H	H···*A*	*D*···*A*	*D*—H···*A*
O4—H4···O2^i^	0.84	1.69	2.521 (3)	171
O8—H8*A*···O6^ii^	0.84	1.71	2.547 (3)	171
O12—H12*A*···O10^iii^	0.84	1.73	2.565 (3)	177
N1—H1···Cl3	0.93	2.46	3.334 (3)	156
N2—H2*C*···Cl6^iv^	0.92	2.45	3.249 (3)	145
N3—H3···Cl2	0.93	2.48	3.354 (3)	156
N4—H4*A*···Cl3	0.92	2.35	3.216 (3)	156
N5—H5···Cl4	0.93	2.44	3.319 (3)	159
N6—H6*D*···Cl1^v^	0.92	2.37	3.235 (3)	156

Symmetry codes: (i) *x*, *y*−1, *z*; (ii) *x*, *y*+1, *z*; (iii) *x*+1, *y*+1, *z*; (iv) *x*+1, *y*, *z*−1; (v) *x*, *y*, *z*+1.
